# Toothpaste ingestion—evaluating the problem and ensuring safety: systematic review and meta-analysis

**DOI:** 10.3389/fpubh.2023.1279915

**Published:** 2023-10-20

**Authors:** Bojan Petrović, Sanja Kojić, Lazar Milić, Alessandro Luzio, Tamara Perić, Evgenija Marković, Goran M. Stojanović

**Affiliations:** ^1^Faculty of Medicine, University of Novi Sad, Novi Sad, Serbia; ^2^Faculty of Technical Sciences, University of Novi Sad, Novi Sad, Serbia; ^3^Istituto Italiano di Tecnologia (IIT) Center for Nano Science and Technology (CNST), Milan, Italy; ^4^Clinic for Pediatric and Preventive Dentistry, School of Dental Medicine, University of Belgrade, Belgrade, Serbia; ^5^Clinic for Orthodontics, School of Dental Medicine, University of Belgrade, Belgrade, Serbia

**Keywords:** toothpaste ingestion, health effects, adverse events, systemic toxicity, active ingredients, public health

## Abstract

This systematic review and meta-analysis aimed to evaluate the ingestion of toothpaste and its sequelae. The study adhered to the PRISMA guidelines and was registered in the PROSPERO database. A comprehensive search strategy was conducted across multiple databases, resulting in the inclusion of 18 relevant publications. Eligible studies encompassed various designs and included both children and adults as the study population. Data extraction was carried out systematically, and relevant information on study characteristics, interventions, and outcomes were collected. The assessment of bias was performed using the Joanna Briggs Institute’s Critical Appraisal Tools showing variations of bias among the included studies. The overall risk of systemic toxicity was found to be low, and no severe or life-threatening events were reported in the included studies. Furthermore, some toothpaste formulations containing higher concentrations of fluoride were associated with an increased risk of dental fluorosis. These findings have several implications for practice and policy. Healthcare providers and dental professionals should emphasize the importance of promoting safe toothpaste use, especially in vulnerable populations such as young children who are more prone to accidental ingestion. Public health campaigns and educational initiatives should aim to raise awareness about appropriate toothpaste usage and the potential risks. In addition, toothpaste manufacturers and regulatory bodies should consider revising guidelines and regulations to ensure the safety of oral care products, including the appropriate concentration of active ingredients. Future research should focus on investigating the long-term effects of toothpaste ingestion, exploring potential interactions between different active ingredients, and evaluating the efficacy of current preventive measures.

## 1. Introduction

The statement coined by Paracelsus: ‘Is there anything that is not toxic?’ is widely recognized. All objects possess toxicity and nothing exists without it. It is only the dosage that dictates whether or not a substance is poisonous (1).

Children and adults are exposed to various substances in their surroundings on a daily basis, including toothpaste, shampoo, soap, and other household products. While these exposures are usually not harmful, they may require analysis to understand the potential risks associated with ingestion of unwanted materials.

Unintentional or unconscious swallowing refers to the act of ingesting substances without intent or awareness, which can occur in various situations, particularly in vulnerable populations such as children and individuals with cognitive impairments (2). It is important to recognize that not all of unintentional ingestion is necessarily harmful or dangerous. The potential hazards associated with ingestion can vary depending on the nature of the materials and the age group of individuals involved. Young children, especially infants and toddlers, may unintentionally ingest unwanted materials due to their natural curiosity and tendency to explore their surroundings by putting objects in their mouths (3). This can include ingesting household products, food packaging materials, or small objects that pose a choking hazard. On the other hand, older children and adults may have a higher tolerance and ability to metabolize or eliminate ingested substances, reducing the potential for harm. Dufour et al. conducted a study with a sample of 549 participants, which was evenly divided by sex and swimmers of both young and adult age groups were included (4). The findings revealed that swimmers ingested an estimated mean of 32 mL of water per hour during swimming activities. Children swallowed approximately four times more water than adults did. However, children spent approximately twice as much time in the water compared to adults. Additionally, males tended to ingest more water than females during swimming, as per the study findings.

Individuals with cognitive impairments or developmental disabilities may also have difficulties understanding the concept of ingestion and may inadvertently swallow unwanted materials due to lack of awareness or impulsivity. Additionally, individuals who are asleep, unconscious, or under the influence of certain medications or substances may inadvertently swallow unwanted materials without realizing it. The severity of potential harm also depend on the quantity, concentration, and duration of exposure. These factors underscore the need for thorough analysis and risk assessment of the materials in question, as well as appropriate preventive measures based on the specific risks identified.

Toothpaste is commonly used by individuals of all age groups as essential components of their daily oral hygiene routine. However, unlike other oral care products and dental devices that are primarily used externally, toothpaste is more likely to be inadvertently swallowed during use. The recently published review on fluoride intake from oral hygiene products application underlines that, regardless of the fluoride concentration in drinking water, dental care products contribute significantly to overall fluoride consumption (5). Depending on factors like concentration, chemical forms, and product usage, the contribution from dental materials and prophylactic agents might vary greatly. To what extent these products contribute to total daily fluoride intake appears to depend on how they are used. Appropriate use of toothpaste allows for the control and individualized adjustment of fluoride intake. The optimal daily fluoride intake recommended by European Food Safety Authority at roughly 50–70 g/kg bw/day may also need to be reevaluated in order to find the optimal daily dental product-derived fluoride intake for each person given the contribution of dental care products to total fluoride intake (6).

The study conducted by Saad et al. demonstrates that when considering the contribution of dental care items, constituting approximately 39–51% of the total intake, the optimal daily dietary fluoride intake might be half of the recommended amount (5). Toothpaste typically contains several ingredients, and while most are safe, when used as directed, swallowing large amounts of toothpaste can potentially lead to health risks. Apart from fluoride toxicity concerns, some components found in toothpaste that may cause toxic effects if ingested in large quantities include the following:

1. Sodium Lauryl Sulfate (SLS) is a surfactant commonly used in toothpaste to create foam and aid in the plaque removal. Ingesting large amounts of SLS can cause gastrointestinal irritation and may lead to symptoms such as nausea, vomiting, and diarrhea (7);
2. Triclosan is an antibacterial agent found in certain toothpaste formulations. While the use of triclosan in toothpaste has declined in recent years, excessive ingestion of triclosan has been associated with potential adverse effects on the endocrine system and antimicrobial resistance (8).
3. Artificial Sweeteners like saccharin, aspartame, or sucralose are used to enhance flavor. These sweeteners are generally considered safe, but some individuals may have sensitivities or allergies to them. Ingesting large amounts of artificial sweeteners can have laxative effects and may cause gastrointestinal discomfort (9);
4. Titanium dioxide Titanium dioxide (TiO2) is a common component in toothpaste due to its chemical inertness, low cost, and availability (10), and is primarily used as a pigment in toothpaste to provide opacity and brightness (11). However, there are concerns regarding its potential toxicity, particularly when it comes to its recent exposure as a potential carcinogen to humans (10). The controversies surrounding titanium dioxide’s toxicity mainly arise from discrepancies between experimental protocols of toxicity assays and their results, leading to debates and ongoing research in this area (10). For now, titanium dioxide is a subject of scientific investigation, and there is no definitive consensus on its safety or toxicity. In summary, toothpaste ingredients such as Sodium Lauryl Sulfate (SLS), triclosan, artificial sweeteners, and titanium dioxide are commonly used but can have potential health concerns, including gastrointestinal irritation, endocrine disruption, and ongoing debates about the toxicity of Titanium Dioxide.

Regulatory bodies, such as the FDA, provide guidance on the safe use of titanium dioxide in different applications, including toothpaste (10). Recent studies investigating transgenerational effects have raised additional concerns regarding the potential impact on populations. These findings reinforce the European Food Safety Authority’s decision to restrict the use of TiO2, emphasizing the need for caution in their application (12); Abrasives such as hydrated silica or calcium carbonate are used to help remove stains and plaque. Excessive or aggressive brushing with abrasive toothpaste can potentially harm tooth enamel; Artificial Colors are often on a list of ingredients in toothpaste to enhance their appearance. Certain artificial colors, such as FD&C Red No. 40 and FD&C Yellow No. 5, have been associated with allergic reactions and hyperactivity in sensitive individuals.

The revised focus on toothpaste in our systematic review, and meta-analysis stems from their significantly higher potential for ingestion compared to other oral care products, pieces of dental materials and orthodontic appliances. This higher likelihood of ingestion can be attributed to several factors. Firstly, toothpaste is typically formulated with pleasant flavors to enhance the overall user experience. These appealing flavors, coupled with the natural inclination to rinse the mouth after brushing, increase the probability of swallowing small amounts of the products. Additionally, children, in particular, may have difficulty spitting out effectively, resulting in unintended ingestion. Considering the widespread use of toothpaste, along with the higher chances of accidental ingestion and associated risks, it becomes crucial to examine the problem comprehensively. By focusing on the ingestion of toothpaste specifically, our systematic review and meta-analysis will provide valuable insights into the extent of the problem, potential health implications, and approaches for prevention.

## 2. Materials and methods

### 2.1. Study design

This study has been conducted as a Systematic review and Meta-analysis of the literature. The protocol has been established in accordance to the newly updated PRISMA statement (13). The study has been registered on the International Prospective Register of Systematic Reviews (PROSPERO) database under the number CRD42023428780. The Joanna Briggs Institute (JBI) Manual has been used to evaluate bias (14).

### 2.2. Eligibility criteria

The eligibility criteria for study inclusion in this systematic review were as follows:

Inclusion Criteria:

Study Population: Clinical studies without age limitation (including both children and adults) as the primary study population.

Interventions: Studies evaluating exposures related to toothpaste swallowing/ingestion.

Comparators: Studies comparing different exposure levels, or variations in toothpaste formulations.

Outcomes: Studies reporting outcomes related to the ingestion of toothpaste, including but not limited to adverse health effects, toxicity, allergic reactions, environmental impact, or safety concerns.

Study Design: Randomized controlled trials (RCTs), non-randomized controlled trials, cohort studies, case-control studies, cross-sectional studies, and observational studies with comparative designs.

Time Frame: Studies published from the earliest available date up to the present.

Report Characteristics: Full-text articles published in peer-reviewed journals.

Exclusion Criteria:

- Studies unrelated to oral hygiene products, oral devices, or similar sources.
- Studies without relevant outcome measures.
- Case reports, opinion articles, editorials, and systematic reviews without original data.
- Abstracts, posters, conference abstracts, dissertations, and unpublished studies.
- Duplicate studies or overlapping datasets.
- Studies with insufficient data or incomplete reporting.

### 2.3. Sources of information and search strategy

The search strategy was carried out on May 26, 2023, and included four databases: PubMed, Google Scholar, Web of Science (WOS), and Cochrane Reviews. The key words used for the search were “Toothpaste swallowing” OR “Toothpaste ingestion” OR “Dentifrice swallowing” OR “Dentifrice ingestion.” The search terms were applied to the title and abstract fields of the articles. The search strategy aimed to identify relevant studies evaluating the exact amount or the proportion of the toothpaste swallowed or ingested during toothbrushing.

To systematically extract pertinent data from the listed research, structured data extraction templates were created. Microsoft Excel spreadsheet software was used to develop these templates. The forms had pre-set fields for important study characteristics, results, and other pertinent information. All review-related documents and data were put in a safe, centralized location. This was accomplished using Google Drive, a cloud-based storage service. All review operations, including search tactics, screening procedures, data extraction techniques, and any adjustments or choices made throughout the study, were meticulously documented.

### 2.4. Study selection

Multiple phases of the study selection procedure were carried out by two independent reviewers (BP and SK). Initially reference management software, Rayyan was used to eliminate duplicate records (15). Next task included assessment of the remaining records’ titles and abstracts in accordance with the predetermined qualifying requirements. Then, the whole texts of possible eligible articles were acquired and reviewed for potential inclusion. Discussion and, if necessary, consultation with a third reviewer (LM) were used to settle any differences or conflicts between the reviewers. Each study was thoroughly reviewed based on the stated eligibility requirements. Regarding their applicability to the specific goals of the review as well as the research issue, the included studies underwent a comprehensive evaluation. The final study selection process was documented, and the exclusion criteria were noted.

### 2.5. Data collection

The full-text articles that satisfied the inclusion criteria were screened for pertinent data during data collecting phase. To ensure accuracy and consistency, the extraction process was carried out in a controlled and systematic way. The authors, the publication year, and the country where the study was conducted were taken out of each included paper. The primary goal of each study was extracted in order to provide a thorough summary. Additionally, a focus was placed on gathering data regarding the study sample. This includes information on the study’s inclusion and exclusion criteria, as well as the total number of participants. Understanding these features was crucial for determining how applicable and generalizable the study’s findings were. The precise amount of toothpaste ingested or the percentage of toothpaste swallowed by participants were recorded as relevant outcome measures in addition to the aforementioned data points.

### 2.6. Bias assessment

The Joanna Briggs Institute’s (JBI) Critical Appraisal Tools were used to assess the risk of bias in the included studies during the bias assessment phase. These methods offered a systematic framework for evaluating the methodological integrity and potential bias-inducing factors in various study designs. Two reviewers independently evaluated each included study using the JBI Critical Appraisal Tool that was appropriate for the particular study design. This strategy guaranteed that the included studies were evaluated thoroughly and systematically. A set of criteria that addressed many facets of study design, conduct, and reporting made up the JBI Critical Appraisal Tools. Based on the data from the study, the reviewers evaluated each criterion and gave a judgment of “Yes,” “No,” “Unclear,” or “Not applicable” for each. A “Yes” response was worth one point, a “No” response was worth zero points, and an “Unclear” response was worth half a point. The final result was then given as a score out of 100. Each checklist domain was evaluated independently by BP and SK, two evaluators. The study which received a score of less than 49% was marked as moderate, a score of 50–69% risk of bias. The study with score of more than 70% was classified as high risk of bias (14, 16). The study design affected the criteria that were assessed during the bias assessment process. To acquire the data required for the bias evaluation, the reviewers thoroughly studied the pertinent sections of each paper, including the methodology, results, and discussion. Discussion and agreement among the reviewers were used to settle any differences or conflicts in their evaluations. When agreement could not be obtained, the final judgment was determined after consulting a third reviewer (LM) (Table 1).

**Table 1:** Cross sectional studies’ risks of bias assessed by checklist for Cross Sectional Studies from Joanna Briggs Institute’s (JBI) Critical Appraisal Tools (17). (1 = yes – 0.5 = unclear – 0 = no – NA = not applicable).

Author	1. Were the criteria for inclusion in the sample clearly defined?	2. Were the study subjects and the setting described in detail?	3. Was the exposure measured in a valid and reliable way?	4. Were objective, standard criteria used for measurement of the condition?	5. Were confounding factors identified?	6. Were strategies to deal with confounding factors stated?	7. Were the outcomes measured in a valid and reliable way?	8. Was appropriate statistical analysis used?	Total	%
Hargreaves et al. (18)	0.5	1	1	0.5	0	0	1	1	5	62.5
Barnhart et al. (19)	0.5	1	1	1	0	0	1	1	5.5	68.8
Naccache et al. (20)	0	0.5	1	0.5	0	0	1	1	4	50
Sjögren et al. (17)	0	1	1	1	0	0	1	1	5	62.5
Bentley et al. (21)	0	0.5	1	1	0	0	0.5	1	4	50
Rojas-Sanchez et al. (22)	0	1	1	0.5	0	0	1	1	4.5	56.3
Puppin Rontani et al. (23)	1	1	1	1	0	0	1	1	6	75
Murakami et al. (24)	0.5	1	1	1	1	1	1	1	7.5	93.8
Paiva et al. (25)	0.5	1	1	1	1	1	1	1	7.5	93.8
Pessan et al. (26)	0.5	1	1	0.5	0.5	0.5	1	1	6	75
Martínez‐Mier et al. (27)	1	1	1	1	1	1	1	1	8	100
Cochran et al. (28)	0.5	1	1	0.5	1	0	1	1	6	75
Cardoso et al. (29)	1	1	1	1	1	0	1	1	7	87.5
Moraes et al. (30)	0	1	0.5	1	0	0	1	1	4.5	56.3
De Almeida (31)	0	1	1	1	1	1	1	1	7	87.5
Kobayashi et al. (32)	0	1	1	1	1	1	1	1	7	87.5
Lima-Arsati et al. (33)	0	0.5	1	1	0.5	0.5	1	1	5.5	68.8
Strittholt et al. (34)	1	1	1	1	1	1	1	1	8	100

In order to determine the statistical power and generalizability of the results, the sample size of each study was examined. The rigor and validity of the research design were evaluated based on the methods used in each study. Aspects like randomization, blinding, allocation concealment, data collection techniques, and follow-up protocols were evaluated during this process. Based on the determined characteristics and bias risk, the overall quality of each study was appraised.

Each included study’s primary findings and characteristics have been condensed into a descriptive summary. In order to do this, relevant information about the studies’ design, participant characteristics, interventions, outcomes, and findings had to be extracted.

A meta-analysis was carried out for the included papers that were deemed suitable for quantitative synthesis and had enough uniformity in terms of research design, treatments, and outcome measures. Depending on the kind of outcome measures and the information provided, specific models were used:

- Calculation of Effect Size: Using data from individual studies, the relevant effect size measure (such as risk ratios, odds ratios, or mean differences) was determined for each outcome of interest.
- Statistical Heterogeneity: The Cochran’s Q test and the I2 statistic were used to examine the statistical heterogeneity among the included studies. Possible sources of heterogeneity were looked at when there was a lot of heterogeneity.
- Forest Plots: Forest plots were used to summarize the meta-analysis’s findings, showing the effect sizes and confidence ranges for each research as well as the combined estimate. With the help of the RevMan (Review Manager) program and the GraphPad prism software suite, statistical analysis and meta-analysis were carried out.

The narrative summaries involved methodically condensing and outlining the pertinent data from each included study, emphasizing the study’s features, interventions, evaluated outcomes, and reported conclusions. The narrative summaries were designed to give a thorough review of the outcomes of each particular investigation, including any variances or discrepancies in findings between studies.

## 3. Results

### 3.1. Study selection

**Figure 1:**
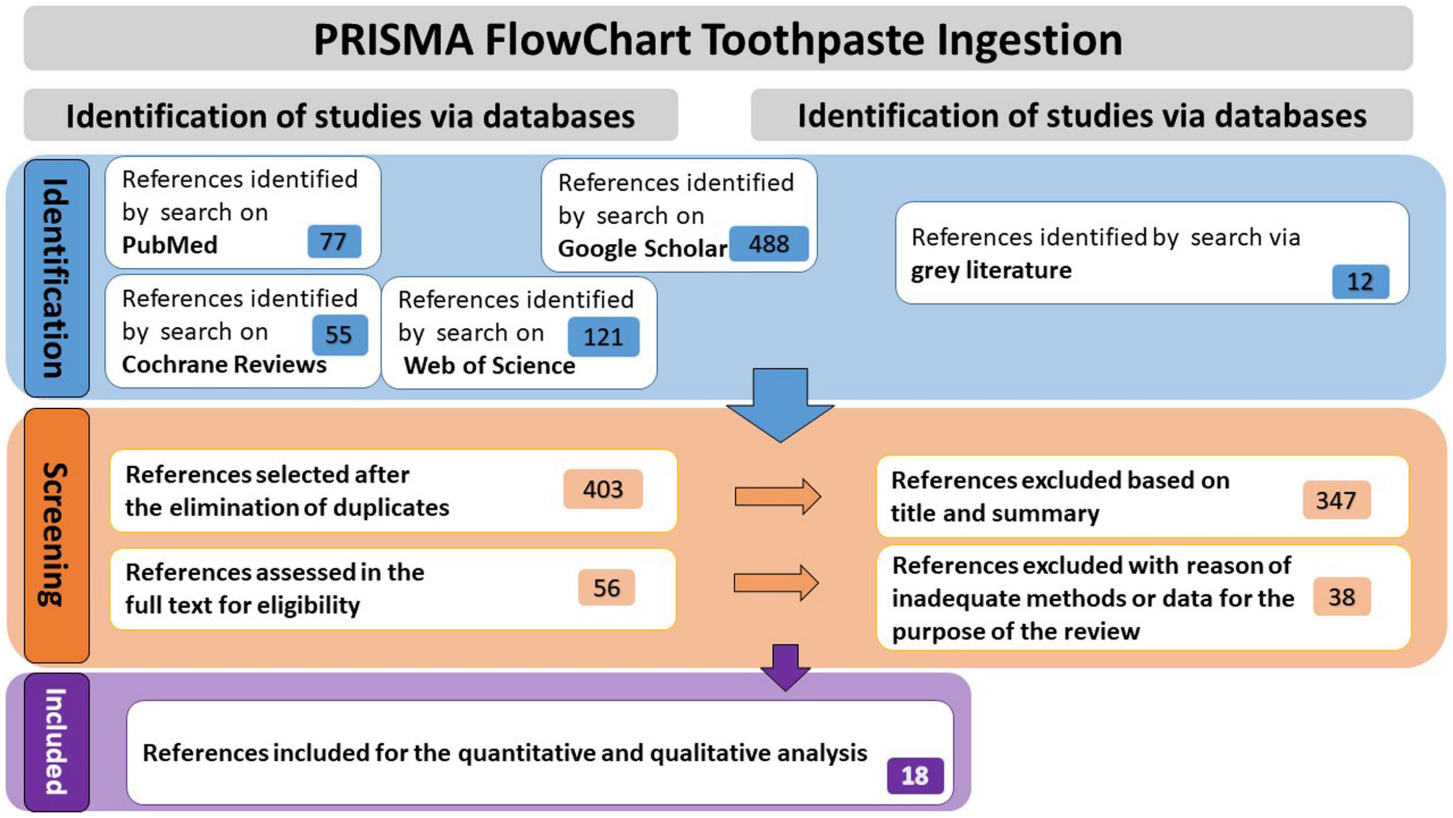
Prisma flowchart of the research process.

Research on four databases (PubMed, Google Scholar, Web of Science, Cochrane Reviews), as well as gray literature, yielded to 753 references. After duplications were eliminated, 403 articles underwent a thorough review of their titles and abstracts. Fifty six of them were chosen for a detailed evaluation of their eligibility criteria, following elimination of 347 articles because they did not correspond with the review’s and meta-analysis’s objectives. In the end, 18 publications that fully complied with established requirements were included in the systematic review and meta-analysis (see Figure 1).

**Figure 2:**
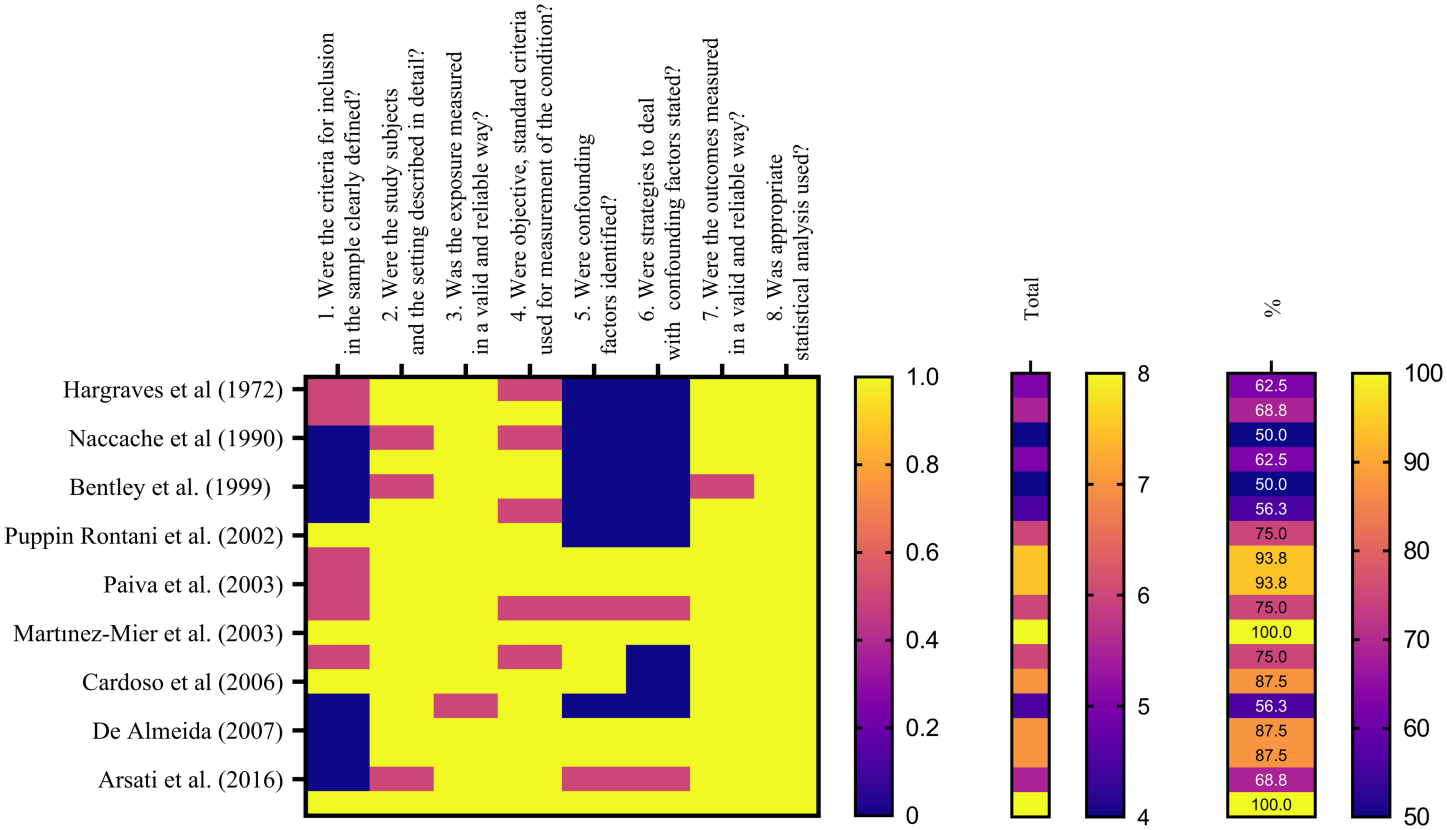
Heat map describing the results of bias assessment.

Table 2 and Figure 2 present the assessment of cross-sectional studies’ risks of bias using the Joanna Briggs Institute’s Critical Appraisal Tools checklist. The tables display the scores assigned to each study based on specific criteria. The results show variations in the risk of bias across the included studies, with total scores ranging from 4 to 8. Higher scores indicate studies with a lower risk of bias. Notably, studies like those by Martínez-Mier et al. and Strittholt et al. achieved the highest scores, signifying a robust methodology and dependable findings. In contrast, some studies received lower scores, implying a higher potential for bias (27, 34). Table 3 provides a summary of toothpaste ingestion data from various studies conducted between 1972 and 2016. The sample sizes ranged from 5 to 700 participants, and the mean percentage of toothpaste ingested varied from 10.66 to 80.54%. The standard deviations (SD) indicate the variability in the data, with values ranging from 0.984 to 56.53. Additionally, the table includes the 95% confidence intervals (CI) for the mean percentage of toothpaste ingested, which provides a range of values within which we can be confident that the true population mean lies. Data evidently show that the mean percentage of toothpaste ingestion varied across the studies. For example, Murakami et al. (24) reported a relatively low mean ingestion rate of 10.66% (24), whereas De Almeida et al. (31) found the highest mean ingestion rate at 80.54% (31). These findings suggest a considerable variability in toothpaste ingestion behaviors among different populations or under varying conditions.

**Table 2:** Summary of findings from clinical studies (number of participants, age, country and main findings related to the toothpaste ingestion).

Study	*N*	Age	Country	Main findings related to the toothpaste ingestion
Hargreaves et al. (18)	44	4–6	CAN	A study on 44 children in Edinburgh found that, on average, 27.60% of toothpaste was ingested. The highest amount swallowed was 1.16 g per brushing. However, this technique may overestimate ingestion
Barnhart et al. (19)	251	2–35	USA	Data on dentifrice ingestion were collected across different age groups (2–4, 5–7, 11–13, and 20–35 years). Ingestion decreased with age, ranging from 0.30 to 0.04 grams per brushing
Naccache et al. (20)	48	3–5	CAN	Children aged 3–5 years to examine the variability in the amount of dentifrice used and ingested. The difference in the amount used between any two brushings was less than 0.250 g Mean % of toothpaste ingested: 35.47
Sjögren et al. (17)	8	26–45	SWE	Eight adults, four experiments. Quick rinses showed minimal absorption, long-lasting rinses and no rinsing resulted in varying fluoride absorption
Bentley et al. (21)	49	2–3	UK	50 children aged 30 months visited at home. 0.36 g of toothpaste was applied, with 72% retained in the mouth. The average fluoride ingestion per brushing was 0.42 mg F toothpaste and 0.10 mg with 400 ppm F toothpaste
Rojas-Sanchez et al. (22)	54	1–3	USA	Intake from diet and dentifrice in three groups of 16- to 40-month-old children. Reducing fluoride intake is necessary in both negligibly and optimally fluoridated communities to prevent dental fluorosis
Puppin Rontani et al. (23)	144	3–9	BRA	This study analyzed the brushing habits and fluoride ingestion in Brazilian children aged 3–9 years. Dentifrice brand significantly affected dentifrice amount placed, amount ingested, fluoride ingestion, and brushing time
Murakami et al. (24)	94	3–5	JAP	Fluoride intakes in 94 preschool children aged 3, 4, and 5 years. The average total fluoride intake from diet and dentifrice for 3- to 5-year-old Japanese children was 0.35 mg/day (0.021 mg/kg body weight)
Paiva et al. (25)	71	1–3	BRA	71 Brazilian children aged 19–38 months, from two fluoridated communities, had their fluoride intake from diet and dentifrice assessed. Daily fluoride intake from dentifrice exceeded intake from the diet in both communities
Pessan et al. (26)	21	4–7	BRA	Mean fluoride intake dentifrice was 0.037 ± 0.038. Dentifrice accounted for 57.43 ± 29.02% of fluoride intake
Martínez‐Mier et al. (27)	39	1–3	MEX	Children had a mean fluoride intake of 0.20 ± 0.08 mg fluoride/kg/day and 0.18 ± 0.07 mg fluoride/kg/day
Cochran et al. (28)	700	1.5–3.5	EU	Significant variation was found in toothpaste types, amounts applied, and amounts ingested. Fluoride ingestion ranged from 0.01 to 0.04 mg/kg
Cardoso et al. (29)	5	25–35	BRA	Fluoride intakes ranged from 0.29 to 3.16 mg/day from dentifrice. Plasma fluoride concentrations were positively associated with dentifrice intake
Moraes et al. (30)	33	2–3	BRA	Intake from dentifrices in children aged 24–36 months. The impact of dentifrice flavor on ingestion was also examined. Approximately 60% of the loaded dentifrice was ingested. Dentifrice flavor did not affect intake
De Almeida (31)	13	1–3	BRA	This study estimated fluoride intake in 1- to 3-year-old children from their diet and dentifrice. Results showed mean dentifrice intake of 0.10 ± 0.085 mg/kg/day
Kobayashi et al. (32)	155	2–6	BRA	Dentifrice ingestion by 155 children aged 2–6 years. Inverse relationship between age and dentifrice ingestion. Amount used also influenced ingestion
Lima-Arsati et al. (33)	26	2–3	BRA	Study included 155 children aged 2 to 6 years. Results showed lower ingestion with increasing age. The amount used also influenced ingestion
Strittholt et al. (34)	90	2–12	USA	Randomized, single-blinded, crossover study, three age groups (2–4 years, 5–7 years, 8–12 years). Results showed that younger children ingested more dentifrice than older children, and dentifrice usage increased with age

**Table 3:** Summary of Toothpaste Ingestion Data for Meta-analysis.

Study	Sample size	Mean % of toothpaste ingested	SD	95% CI	Cohen’s d
Hargreaves et al. (18)	44	27.60	19.30	21.45–33.75	−0.38
Barnhart et al. (19)	251	18.98	31.24	−3.13 to 41.09	0.61
Naccache et al. (20)	48	35.47	21.87	26.30–44.64	0.02
Sjögren et al. (17)	8	20.33	17.38	−4.91 to 45.57	1.17
Bentley et al. (21)	49	72.00	17.00	67.24–76.76	3.23
Rojas-Sanchez et al. (22)	54	21.48	4.70	20.05–22.91	4.57
Puppin Rontani et al. (23)	144	34.00	19.51	30.42–37.58	1.74
Murakami et al. (24)	94	10.66	1.20	10.42–10.89	0.55
Paiva et al. (25)	71	60.83	18.56	62.14–71.26	−0.20
Pessan et al. (26)	21	34.09	19.07	25.60–42.58	1.79
Martínez‐Mier et al. (27)	39	69.50	46.85.	54.58–84.42	1.48
Cochran et al. (28)	700	73.00	8.42	72.15–73.58	8.66
Cardoso et al. (29)	5	36.36	29.46	−4.02 to 76.74	1.23
Moraes et al. (30)	33	60.94	2.43	59.68–62.20	25.12
De Almeida (31)	33	80.54	56.53	68.53–92.56	1.42
Kobayashi et al. (32)	155	66.80	1.55	66.57–66.93	−3.92
Lima-Arsati et al. (33)	26	70.50	24.00	59.43–81.57	2.94
Strittholt et al. (34)	90	53.00	0.98	52.80–53.19	3.39
Tau^2^	3.680				
Chi^2^	216.216				
Df	17.000				
*P*	0.005				
*I* ^2^	93.875				
*Z*	3.115				
*P*	0.0009				

Additionally, standard deviations signify the dispersion of data points around the mean. Studies such as the one conducted by Cochran et al. (28) and De Almeida et al. (31) reported larger standard deviations, indicating greater variability in toothpaste ingestion within their respective samples. In contrast, smaller standard deviations, suggesting less variation in toothpaste ingestion behavior, were reported in studies by Murakami et al. (24) and Strittholt and his associates (34).

**Figure 3:**
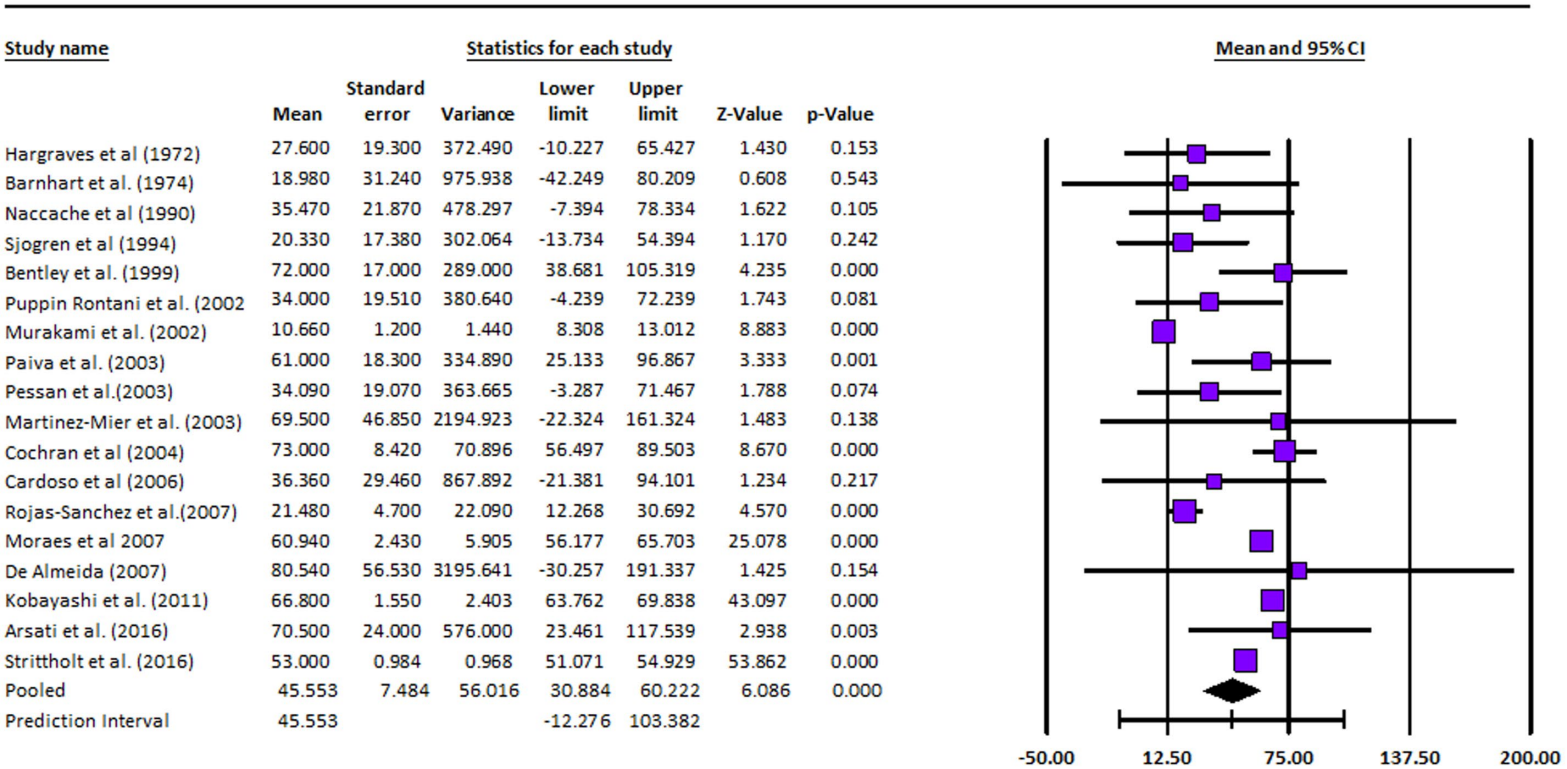
Forest plot: meta-analysis results.

Effect sizes, as measured by Cohen’s d, varied across the studies, indicating diverse magnitudes of the association between toothpaste ingestion and the investigated factors. Studies such as the one by Cochran et al. (28) and the study conducted by Moraes and team (30) notably reported significantly higher effect sizes, indicating a strong relationship between toothpaste ingestion and the associated variables. In contrast, the research by Hargreaves et al. (18) and the study led by Paiva et al. (25) demonstrated smaller effect sizes, suggesting a weaker association (18, 25). The confidence intervals (CIs) of the effect sizes varied across studies, highlighting the need for caution in generalizing these findings. Furthermore, the statistical analysis revealed a significant heterogeneity among the included studies, as indicated by a high *I*^2^ value of 93.875% and a significant chi-square value (χ^2^ = 216.216, df = 17, *p* = 0.005). This suggests that factors beyond chance alone contribute to the observed variability among the studies. Consequently, it is crucial to consider the influence of these factors when interpreting the overall findings of the meta-analysis. In summary, the meta-analysis findings indicate a wide range of toothpaste ingestion percentages across the included studies. The effect sizes varied, suggesting differing strengths of the relationship between toothpaste ingestion and associated variables. The significant heterogeneity observed underscored the importance of accounting for potential contributing factors (Figure 3).

The analysis was based on 18 studies. The effect size index was the mean. The random-effects model was employed for the analysis. The studies in the analysis were assumed to be a random sample from a universe of potential studies, and this analysis was used to make an inference to that universe (35–50). The mean effect size is 45.553 with a 95% confidence interval of 30.884–60.222. The mean effect size in the universe of comparable studies could fall anywhere in this interval. The *Z*-value tests the null hypothesis that the mean effect size was zero. The *Z*-value is 6.086 with *p* < 0.001. Using a criterion alpha of 0.050, we rejected the null hypothesis and concluded that in the universe of populations comparable to those in the analysis, the mean effect size was not precisely zero. The Q-statistic provided a test of the null hypothesis that all studies in the analysis shared a common effect size. If all studies shared the same true effect size, the expected value of *Q* would be equal to the degrees of freedom (the number of studies minus 1). The *Q*-value is 1162.965 with 17 degrees of freedom and *p* < 0.001. Using a criterion alpha of 0.100, we could reject the null hypothesis that the true effect size was the same in all these studies. In this case the *Q*-value is 1162.965 with 17 degrees of freedom and *p* < 0.001. Using a criterion alpha of 0.100 we could reject the null hypothesis that the true effect size was the same in all these studies. The I-squared statistic was 99%, which informed us that some 99% of the variance in observed effects reflected variance in true effects rather than sampling error. Tau-squared, the variance of true effect sizes was 688.134 in raw units. Tau, the standard deviation of true effect sizes, was 26.232 in raw units. Computations were carried out using Comprehensive Meta-Analysis Version 4 (35–40).

## 4. Discussion

The various percentages of toothpaste ingestion have been a consistent finding in the literature. These substantial variations could be attributed to several factors. Firstly, differences in study populations, socio-economic status, age ranges and geographical locations, may have contributed to reported discrepancies. Secondly, variations in dentifrice brands, fluoride concentrations, and brushing habits may have been accountable for the observed differences. Hargreaves et al. discovered an average toothpaste ingestion of 27.60% in a study involving 44 children in Edinburgh, with the highest amount swallowed reaching 1.16 grams per brushing (18). In contrast, Barnhart and team collected data on dentifrice ingestion across different age groups in the USA and observed a decrease in ingestion with age, ranging from 0.30 to 0.04 grams per brushing (19). Studies conducted in Canada (20) and Brazil (23) reported 35.47% and approximately 60% of loaded dentifrice ingested, respectively. Pessan et al. found that dentifrice intake accounted for 57.43 ± 29.02% of fluoride ingestion in a study involving Brazilian children aged 4–7 years old (26). Furthermore, the influence of age on toothpaste ingestion has been consistently demonstrated, with younger children tending to ingest more toothpaste than older children (34–36). Younger children have limited ability to spit out excess toothpaste, and propensity to swallow toothpaste during brushing. These findings underscore the importance of age-appropriate guidelines for toothpaste usage and supervision during brushing routines and highlight the potential risk of overexposure or inadequate fluoride consumption, jeopardize caries prevention efforts and overall health. In studies conducted in Japan (24) and Mexico (27), the average total fluoride intake from diet and dentifrice ranged from 0.35 to 0.20 ± 0.08 mg fluoride/kg/day, respectively. Rojas-Sanchez et al. emphasized the need to reduce fluoride intake in both negligibly and optimally fluoridated communities to prevent dental fluorosis in young children (22). Authors were guided by the significant concern is the adverse effects of ingested fluoride, particularly in excessive amounts. Fluoride is an essential component in toothpaste for its preventive effects against dental caries. However, excessive fluoride ingestion can lead to dental fluorosis, which manifests as white or brown stains on the teeth. Adhering to recommended dosage guidelines, such as using a pea-sized amount of toothpaste for children under 6 years old, is crucial in mitigating the risk of excessive ingestion and associated oral and general health complications. It is important for dental professionals, parents, and caregivers to be aware of the potential consequences of excessive toothpaste ingestion and to promote safe and appropriate toothpaste use among children.

Socio-economic status could potentially play an important role in toothpaste ingestion patterns. Children from lower socio-economic backgrounds may have limited access to oral health information and resources, which can contribute to improper toothpaste use and higher ingestion rates. Cultural practices and beliefs can influence toothpaste ingestion as well. In some cultures, toothpaste is perceived as a food product or a remedy for various ailments, leading to higher ingestion rates. Conversely, in communities with a strong oral health awareness culture, individuals may be more cautious, and adhere to recommended toothpaste usage guidelines. Understanding these factors is crucial for developing targeted interventions and education campaigns to promote safe and appropriate toothpaste use among different populations. In addition to individual and cultural factors, packaging, product formulation, and marketing strategies also play a role in toothpaste ingestion behaviors. The design and functionality of toothpaste such as the flavor, and texture of toothpaste, can affect the overall experience and satisfaction during brushing, and influence the dispensed amount, potentially inducing ingestion behaviors. Eye-catching packaging designs, promotional messages, and endorsement by dental professionals can influence product selection and consumer habits, including the amount of toothpaste dispensed and potentially ingested. Child-resistant caps and clear dosage instructions on packaging can help mitigate ingestion risks, particularly for young children.

The main objective of this review article and meta-analysis was to examine the potential health effects and risks associated with toothpaste ingestion, particularly among children. Our analysis of 18 relevant studies revealed important findings regarding toothpaste ingestion and its implications for health. The majority of toothpaste-related incidents involved children under the age of six, highlighting the importance of parental supervision during brushing to prevent accidental ingestion. The present review identified excessive fluoride intake as the primary concern when it comes to toothpaste ingestion. While fluoride is effective in preventing dental caries, excessive ingestion can lead to fluorosis, a condition characterized by dental enamel discoloration and, in severe cases, skeletal fluorosis. Comparing our findings with previous studies, our results are consistent with the existing body of evidence. Our meta-analysis further strengthens this evidence by synthesizing data from multiple studies, allowing for a more comprehensive evaluation of the potential health effects.

The identification of fluoride as a primary concern reinforces the need for further investigation and targeted interventions in this area. From a theoretical standpoint, these findings support and extend existing theories and concepts related to oral health, pediatric medicine, and public health. The study contributes to our understanding of the potential health risks associated with routine oral hygiene practices and emphasizes the importance of considering factors beyond dental caries prevention. It underscores the need to explore the broader systemic effects of toothpaste ingestion, such as the impact on neurodevelopment, endocrine function, and gastrointestinal health. This knowledge can inform future theoretical frameworks and research models, encouraging a holistic approach to oral health promotion and management. In terms of practice, the findings of present study have immediate implications for healthcare professionals, oral health providers, and parents. Healthcare professionals should integrate these findings into their clinical practice and patient education efforts. This information can help practitioners tailor their recommendations to ensure safe oral hygiene practices for children. Furthermore, these findings can guide the development of preventive strategies and interventions aimed at minimizing the risk of toothpaste ingestion. Dental and public health organizations can use this evidence to design educational campaigns targeting both healthcare professionals and the public.

Additionally, the findings may influence the formulation and labeling of toothpaste products, prompting manufacturers to provide clearer information, warnings, and safety guidelines on packaging, not only regarding the fluoride, but all other components, as well. Regulatory authorities can consider incorporating the findings into existing policies or creating new evidence-based guidelines to ensure standardized recommendations for toothpaste formulation, labeling, and advertising. The integration of these research findings into policy development can help protect the health and well-being of vulnerable populations, especially young children. Overall, the review article and meta-analysis findings have far-reaching implications across theory, practice, and policy development in the field of toothpaste ingestion. By advancing our understanding of the associated risks and highlighting the importance of preventive measures, these findings pave the way for improved oral health outcomes and the development of targeted interventions to mitigate the potential adverse effects of toothpaste ingestion.

The present study exhibit several notable strengths. Primarily, this approach minimized the risk of publication bias and ensured a robust foundation for the meta-analysis. By systematically searching multiple databases and including both published and unpublished studies, the review article maximized the inclusiveness of the evidence base. Secondly, the methodology employed in this study was rigorous. The use of predefined inclusion and exclusion criteria helped maintain consistency and transparency in the study selection process. The quality valuation of included studies, such as the risk of bias assessment, enhanced the reliability of the findings. Furthermore, the meta-analysis applied appropriate statistical techniques to synthesize the data and derive meaningful conclusions. The meticulous methodology provided credibility to the study’s findings and strengthened its overall validity. Additionally, the review article explored a large sample size. By including a substantial number of studies and participants, the study achieved a higher statistical power, and allowed more defined estimates of the associations under the investigation. A large sample size enhanced the generalizability of the findings, and increased the confidence in the observed effects. However, certain limitations exist and should be recognized. One potential limitation is the presence of heterogeneity among the included studies. Variations in study design, participant characteristics, outcome measures, and other factors could have introduced heterogeneity, which may have affected the validity of the pooled results.

Another limitation is the potential for biases inherent in the included studies. While scrupulous quality assessment criteria were applied in the review article, the presence of selection bias, measurement bias, or reporting bias in individual studies may have introduced bias into the meta-analysis. These biases could have made an impact on the accuracy and generalizability of the overall findings. Sensitivity analyses and subgroup analyses might help explore the impact of potential biases and provide insights into their influence on the results. Furthermore, the availability and quality of data in the included studies may have posed limitations. We emphasize the importance to acknowledge these limitations as they provide opportunities for future research designed to address the gaps and challenges identified in the current study.

This analysis illuminated potential causal pathways between toothpaste ingestion and health effects, though further exploration is necessary to pinpoint exact mechanisms, This understanding is vital for shaping preventive strategies and public health policies regarding toothpaste usage. While the review established an association, causation remains intricate due to factors such as confounding variables and bias. Caution is needed when interpreting the findings and drawing causal conclusions. Future prospective studies, randomized controlled trials, and mechanistic inquiries to affirm, delve into mechanisms, and establish causal links are needed.

This review and meta-analysis offer thorough insights into existing evidence of toothpaste ingestion’s potential health repercussions, underlining its significance for public health authorities, dental professionals, and policy makers. Dental practitioners are advised to acknowledge risks tied to excessive ingestion and provide prudent recommendation to their patients. Manufacturers of oral care products, public health agencies, and dental professionals should collaborate in promoting safe and appropriate toothpaste use.

## Data availability statement

The original contributions presented in the study are included in the article/supplementary material, further inquiries can be directed to the corresponding author.

## Author contributions

BP: Data curation, Formal analysis, Investigation, Methodology, Writing – original draft. SK: Conceptualization, Data curation, Investigation, Project administration, Visualization, Writing – original draft, Writing – review & editing. LM: Data curation, Formal analysis, Investigation, Methodology, Writing – original draft. AL: Conceptualization, Investigation, Methodology, Supervision, Validation, Writing – original draft. TP: Conceptualization, Formal analysis, Methodology, Validation, Writing – review & editing. EM: Formal analysis, Investigation, Methodology, Validation, Writing – review & editing. GS: Conceptualization, Data curation, Methodology, Project administration, Resources, Supervision, Writing – review & editing.
